# Disinhibited eating mediates differences in attachment insecurity between bariatric surgery candidates/recipients and lean controls

**DOI:** 10.1038/ijo.2017.157

**Published:** 2017-08-08

**Authors:** L L Wilkinson, A C Rowe, C Sheldon, A Johnson, J M Brunstrom

**Affiliations:** 1 Department of Psychology, Swansea University, Swansea, UK; 2 School of Experimental Psychology, University of Bristol, Bristol, UK; 3 Southmead Hospital, North Bristol NHS Trust, Bristol, UK

## Abstract

Previous research has shown that attachment anxiety is a good predictor of body mass index. This relationship is significantly mediated by disinhibited (over-) eating and is likely to reflect a specific form of affect regulation. This study explored whether obese bariatric surgery candidates (BSC; *N*=34) and bariatric surgery recipients (BSR; *N*=15) would show higher levels of attachment insecurity (higher attachment anxiety and/or higher attachment avoidance) than a group of age and gender-matched lean controls (*N*=54). Mediation analyses showed that compared to lean controls (M=2.96, SE=0.1), both BSC (M=3.5, SE=0.2) and BSR (M=3.4, SE=0.2) groups had a more insecure attachment orientation. These relationships were significantly mediated by disinhibited eating (BSC: lower limit confidence interval (LLCI)=0.06 and upper limit confidence interval (ULCI)=0.62; BSR: LLCI=0.02 and ULCI=0.76). There was no such relationship when the BSC and BSR groups were compared (LLCI=−0.15 & ULCI=0.3). These observations suggest that attachment insecurity may be a risk factor for obesity and bariatric surgery because of associated disinhibited eating. Moreover, these factors may be important to consider when bariatric surgery results in poor outcomes.

## Introduction

‘Attachment orientation’ reflects an individual’s expectations and beliefs about themselves and their interpersonal relationships. It tends to be conceptualised in terms of two orthogonal dimensions, attachment anxiety (fear of abandonment) and attachment avoidance (fear of intimacy). Low levels of attachment anxiety and avoidance are associated with a secure attachment orientation and higher levels on either/ both are associated with an insecure attachment orientation.^1^ Attachment orientation is generally thought to be abstracted from early interactions with caregivers.^2^ Attachment security is thought to result from consistent and responsive caregiving, while insecure attachment orientations are thought to result from inconsistent caregiving (attachment anxiety) or caregiver rejection (attachment avoidance).^3^ Notably, attachment orientation is thought to endure into adulthood and be generally stable over time, with only a few exceptions (for example, parental suicide).^4^

Importantly, previous research has shown that attachment 'anxiety' is a good predictor of body mass index (BMI).^5^ This relationship is significantly mediated by disinhibited eating (general propensity to engage in overeating) and is likely to reflect a specific form of affect regulation; individuals who are high in attachment anxiety tend to be poorer at regulating their emotions/stress and are more likely to rely on external sources of affect regulation such as food.^6^ In other words, in the absence of sufficient social reinforcement, food is used instead to manage emotion/ mood.

Consistent with this finding, recent research has shown that attachment anxiety is associated with measures of binge eating,^7^ emotional eating^8^ and higher BMI (than reference group)^9^ in bariatric surgery candidates (BSCs). In addition, following gastric bypass surgery, attachment anxiety has been shown to be associated with poor dietary adherence and less weight loss.^10^

To date, no studies have directly compared attachment anxiety *across* the different participant clusters described in the research mentioned above (that is, lean, obese BSCs and bariatric surgery recipients (BSRs)). Moreover, it is unclear whether differences observed across groups may be accounted for by disinhibited eating (mediation). Understanding the relationship between attachment anxiety and disinhibited eating in BSCs compared with BSRs is relevant from a clinical perspective because lower disinhibited eating scores are associated with greater weight loss following surgery.^11^

Therefore, the present study compared the attachment anxiety and disinhibited eating of a group of BSCs, BSRs (gastric band) and a lean age- and gender-matched control group. First, we hypothesised that the attachment anxiety and disinhibited eating scores of a lean control group would be significantly lower than BSC and bariatric recipient groups. Second, we hypothesised that disinhibited eating would significantly mediate group differences in attachment anxiety.

## Methods

### Participants

Participants (*N*=108) volunteered for the study. BSCs and recipients were recruited via clinics within the Diabetes and Endocrinology Department at Southmead Hospital (North Bristol NHS Trust, UK). Lean age and gender-matched control participants were recruited via the research team’s volunteer database which includes staff, students and members of the community local to the University of Bristol, UK. To meet our inclusion criteria, clinical participants had to be planning to undergo bariatric surgery and have a current BMI of over 30 kg/m^2^ OR to have already undergone bariatric surgery and to have had a pre-surgical BMI of over 30 kg/m^2^. Control participants had to have a BMI between 18 and 25 kg/m^2^ and match (as closely as possible) the gender and age of one of our clinical participants. All participants had to be over the age of 18 years old. Eligible participants were given a letter inviting them to take part in the study by a member of the direct care team (potential bariatric candidates/ recipients) or the research team (potential control participants).

Five participants were excluded from our analyses due to incomplete questionnaires (*n*=2), unknown bariatric group (*n*=2), and the use of gastric bypass rather than a gastric band (*n*=1). Therefore, 103 participants’ data were included in our analyses (34 BSCs, 15 BSRs and 54 participants who were lean but otherwise age- and gender-matched to the bariatric groups). The BSRs were on average 3.1 years post-surgery (s.d.=1.5). Ethical approval was provided by the local Faculty of Science Ethics committee and by the local NHS Research Ethics Committee (South West 4 REC: 10/H0102173).

### Materials

Following Wilkinson *et al.*,^5^ attachment orientation was quantified using the 36-item Experiences in Close Relationships questionnaire.^1^ This comprises two 18-item subscales; attachment anxiety (Cronbach’s *α*=0.9) and attachment avoidance (Cronbach’s *α*=0.91). On a seven-point scale ranging from ‘disagree strongly’ (1) to ‘agree strongly’ (7), participants rated their level of agreement with statements about their experiences of interpersonal relationships. Also following Wilkinson *et al.*,^5^ disinhibited eating was assessed using the 16-item disinhibition subscale (Cronbach’s *α*=0.79) of the Three Factor Eating Questionnaire.^12^ Items on this subscale refer to overeating and loss of dietary control, and responses included true/false categories and ratings on a five-point scale (never (0) to always (4)). Finally, as a potential control measure and following Aarts *et al.*,^10^ we also measured depression and anxiety using the hospitalised anxiety and depression scale (HADS).^13^

### Anthropometric measurements

For the lean control group, participants’ height and weight were recorded by an experimenter and used to calculate BMI. For the BSC and recipient groups, height and up to date weight information was obtained from medical records and used to calculate BMI.

### Procedure

BSCs and recipients were approached by the direct care team at a local diabetes and endocrinology clinic. Volunteers were provided with a ‘participant invitation pack’ which contained a letter of invitation, a participant information sheet, an informed consent form and a stamped addressed envelope. Participants were instructed to return the signed informed consent form to the direct care team if they wanted to participate in the study. Upon receiving a completed consent form, participants were sent a numbered questionnaire pack and their height and weight information was recorded against that number. The completed questionnaire packs were returned to the research team who then matched the questionnaire data to the height and weight information. Debrief sheets were provided at the reception of the clinic and were also available upon request from the research team at any time.

### Data analysis

Preliminary analysis showed that attachment anxiety and attachment avoidance were significantly correlated (*r*=0.5, *P*<0.001). To avoid multicollinarity we created a unidimension^14^ called ‘attachment orientation’ by averaging the attachment anxiety and attachment avoidance dimension scores; this assesses security/insecurity more generally (Cronbach’s *α*=0.93).

Mediation analysis with logistic regression was conducted using PROCESS v2.16.^15^ This approach was selected because it allows for the estimation of a potential indirect pathway, whereby attachment orientation influences the tendency to engage in disinhibited eating and, in turn, this predicts whether a participant is a member of either our lean control group or one of our bariatric surgery groups (candidates or recipients). For an overview of mediation analysis and its application the reader is referred to Hayes.^16^

Three mediation models were conducted with participant group as a dichotomous outcome measure (lean/ bariatric candidate, lean/ bariatric recipient and bariatric candidate/ recipient). For all models, attachment orientation was the predictor variable (higher scores indicate attachment insecurity) and disinhibited eating was the mediator variable. This approach allowed for the estimation of potential direct relationships between these variables and the indirect pathway from attachment orientation to participant group via disinhibited eating. A significant indirect pathway is inferred if the lower and upper limit confidence intervals (LLCI and ULCI, respectively) do not cross zero.

## Results

### Preliminary analyses

As expected, there was a significant main effect of BMI (*P*<0.001, *η*_*p*_^*2*^=.82) with *post-hoc* testing (Bonferroni paired comparisons) showing significant differences between every group. There was a significant main effect of disinhibited eating (*P*<0.001, *η*_*p*_^*2*^=.18) with *post-hoc* testing showing significant differences between the control group and both bariatric surgery groups, but no significant different between bariatric surgery groups. There was also a significant main effect of attachment orientation (*P*=0.045, *η*_*p*_^*2*^=.06) with post-hoc testing showing a significant difference between the control group and the BSC group. There were no significant differences in age, gender or HADS scores (*P*>0.05, *η*_*p*_^*2*^<0.04) across groups. See Table 1 for means, SEs and results of *post-hoc* testing.

### Mediation analyses

**Figure 1:**
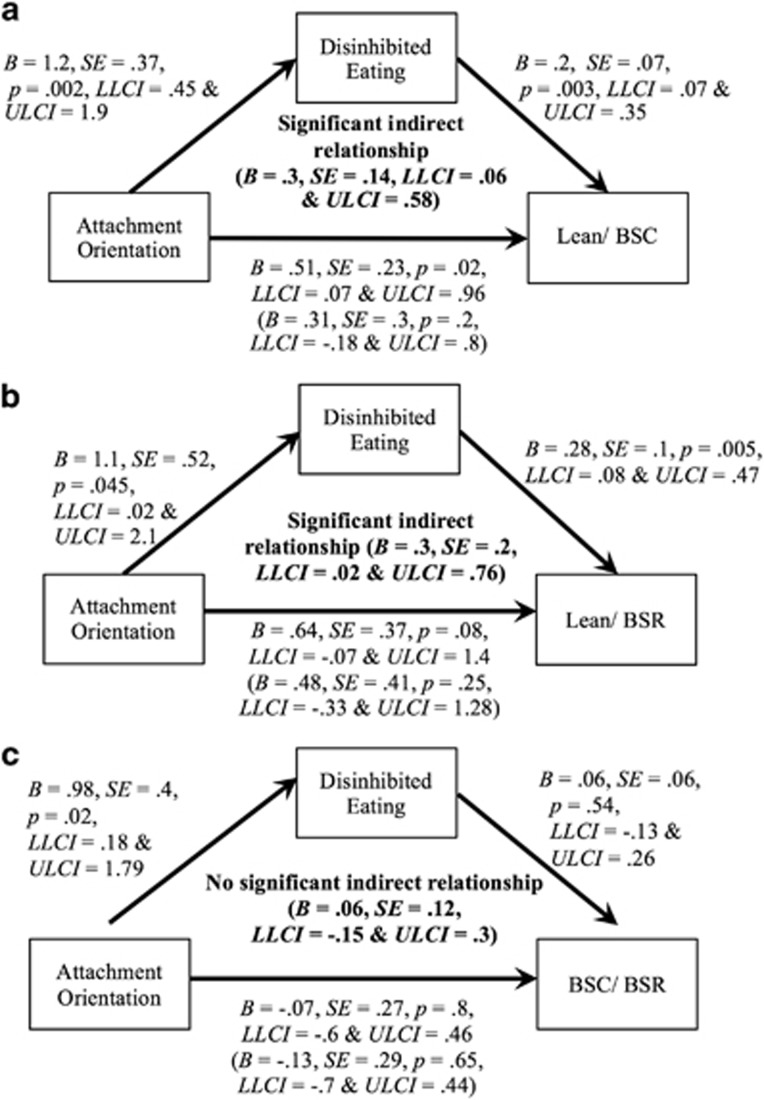
(**a**–**c**) Unstandardised *B* values, SE, *P*-values, LLCI and ULCI are shown for direct effects, values in brackets are direct effects when the mediator is included in the model. LLCI and ULCI are shown for the indirect pathway from predictor to outcome via the mediator. Cox and Snell *R*^*2*^ was calculated for each model (model a=0.16; model b=0.05; model c=0.009).

The first of our mediation models (Figure 1a) showed that attachment orientation significantly predicted participants’ membership of the lean or BSC groups via disinhibited eating (significant indirect pathway). The second of our mediation models (Figure 1b) showed that attachment orientation significantly predicted participants’ membership of the lean or BSR groups directly, and indirectly via disinhibited eating. Finally, our third mediation model (Figure 1c) showed that attachment orientation did not significantly predict participants’ membership of the BSC or BSR groups either directly or indirectly via disinhibited eating. However, a significant relationship between attachment orientation and disinhibited eating was still observed across the BSC/ BSR groups.

## Discussion

Consistent with our hypotheses, individuals in both bariatric groups had significantly higher levels of attachment insecurity than individuals in the lean control group. These relationships were significantly mediated by disinhibited eating. BSRs and candidates had similar attachment orientation scores and were just as likely to engage in disinhibited eating as each other. Generally, attachment insecurity predicted disinhibited eating within these groups. It is likely that despite having already received bariatric surgery, these individuals continued to be poor at affect regulation and continued to manage their emotion by overeating. Indeed, attachment orientation may be a factor worth considering when addressing disinhibited eating, especially in the context of poor outcomes following bariatric surgery.

However, we note that within our significant mediation models, a small amount of the variance associated with differences across groups was accounted for by our predictor (attachment orientation) and mediator (disinhibited eating) variables (16% in the case of model ‘a’ and 5% in the case of model ‘b’). This is consistent with previous studies which have shown modest relationships between psychological traits and BMI.^17^ In addition, our sample was on average 3.1 years post bariatric surgery when poorer outcomes are more likely to be evident.^18^ This study was underpowered to explore whether there was a direct relationship between weight-loss following bariatric surgery and attachment orientation (accounting for time post-surgery); however, this should be considered in future research.

In our sample, attachment anxiety and avoidance scores were significantly correlated. Concomitant high scores on both attachment avoidance and anxiety is known as ‘disorganised attachment’/ ‘fearful avoidance’^19^ and is associated with an increased risk of a clinical diagnoses.^20^ It may be relevant that emerging evidence suggests attachment avoidance is associated with reduced quality of life in BSCs (relative to control)^21^ and poorer appointment attendance following bariatric surgery.^22^ One possibility is that BSRs engage in poor affect regulation strategies (that is, overeating) whilst simultaneously disengaging with their post-surgery care team, and that these work in tandem to limit the benefits of this weight-loss intervention.

Importantly, there is research suggesting that attachment orientation can be temporarily shifted towards attachment security through ‘security priming’. Security priming involves the activation of internal working models associated with attachment security and is associated with a range of positive outcomes.^23^ Some evidence suggests that priming attachment anxiety results in greater intake of a snack food than priming attachment security.^24^ The effect in bariatric surgery patients remains unclear and a study of this kind might provide further evidence for a causal relationship between attachment and dietary relapse.

More generally, future studies might address the limitations associated with the current study. Firstly, this study was cross-sectional in nature and our interpretation of these data rely on previous literature which suggests that attachment orientation is established in early childhood and endures into adulthood. However, an explanation around reverse-causality (that is, that weight category causes poor interpersonal functioning and that this relationship is mediated through disinhibited eating) or an unmeasured additional variable cannot be ruled out. Alternatively, a longitudinal design would allow for such conclusions around causality to be directly tested. Secondly, the current study relied on self-report measures of attachment orientation and disinhibited eating. Future studies might consider alternative assessments of these traits (for example, the adult attachment interview and a laboratory-based eating assessment). Thirdly, the statistical approach here (mediation analysis with logistic regression), while appropriate, precluded us from estimating the amount of variance that the indirect pathway specifically accounted for in our model (rather the reported Cox and Snell *R*^*2*^ values reflected overall variance accounted for by our models). As statistical approaches of this kind advance, this is a question that a future study may pursue. Finally, the assessment of attachment in participants who have undergone alternative and more recently developed surgical procedures, such as a vertical sleeve gastrectomy might be considered.

## Figures and Tables

**Table 1:** Sample size (*N*), mean and SE for age, BMI, HADS total score, disinhibition score and attachment orientation score are reported for each group

*Group*	*N*	*Mean age (years)*	*Mean BMI (kg/m*^*2*^)	*Sex (M/F)*	*HADS total score*	*Disinhibition score*	*Attachment orientation score*
Lean	54	48.5 (SE=1.3)^a^	23.1 (SE=0.2)^a^	15/39	22 (SE=0.35)^a^	5.2 (SE=0.5)^a^	2.96 (SE=0.1)^a^
BSC	34	46.5 (SE=1.5)^a^	48.6 (SE=1.4)^b^	8/26	22.5 (SE=0.56)^a^	8.1 (SE=0.6)^b^	3.5 (SE=0.2)^b^
BSR	15	52.3 (SE=2.8)^a^	40.4 (SE=2.3)^c^	5/10	22.6 (SE=0.7)^a^	8.7 (SE=0.8)^b^	3.4 (SE=0.2)^a,b^

Abbreviations: BMI, body mass index; BSC, bariatric surgery candidates; BSR, bariatric surgery recipients; HADS, hospitalised anxiety and depression scale.

The proportion of male and female participants per group is also reported. Per column, different superscript letters denote significant differences (*P*<0.05) across groups.

## References

[bib1] Brennan KA, Clark CL, Shaver PR. Self-report measurement of adult attachment: An integrative overview. Guildford Press: New York, NY, USA, 1998.

[bib2] Bowlby J. Attachment and Loss: Attachment; John Bowlby. Basic Books: New York, NY, USA, 1969.

[bib3] Bowlby J. Attachment and loss: retrospect and prospect. Am J Orthopsychiatry 1982; 52: 664. 7148988 10.1111/j.1939-0025.1982.tb01456.x

[bib4] Waters E, Merrick S, Treboux D, Crowell J, Albersheim L. Attachment security in infancy and early adulthood: A twenty‐year longitudinal study. Child Dev 2000; 71: 684–689. 10953934 10.1111/1467-8624.00176

[bib5] Wilkinson LL, Rowe AC, Bishop RJ, Brunstrom JM. Attachment anxiety, disinhibited eating, and body mass index in adulthood. Int J Obes (Lond) 2010; 34: 1442–1445. 20351734 10.1038/ijo.2010.72

[bib6] Maunder RG, Hunter JJ. Attachment and psychosomatic medicine: developmental contributions to stress and disease. Psychosom Med 2001; 63: 556–567. 11485109 10.1097/00006842-200107000-00006

[bib7] Shakory S, Van Exan J, Mills JS, Sockalingam S, Keating L, Taube-Schiff M. Binge eating in bariatric surgery candidates: the role of insecure attachment and emotion regulation. Appetite 2015; 91: 69–75. 25828596 10.1016/j.appet.2015.03.026

[bib8] Taube-Schiff M, Van Exan J, Tanaka R, Wnuk S, Hawa R, Sockalingam S. Attachment style and emotional eating in bariatric surgery candidates: the mediating role of difficulties in emotion regulation. Eat Behav 2015; 18: 36–40. 25875114 10.1016/j.eatbeh.2015.03.011

[bib9] Pratt KJ, Balk EK, Ferriby M, Wallace L, Noria S, Needleman B. Bariatric surgery candidates’ peer and romantic relationships and associations with health behaviors. Obes Surg 2016; 26: 2764–2771. 27143096 10.1007/s11695-016-2196-y

[bib10] Aarts F, Geenen R, Gerdes VE, van de Laar A, Brandjes DP, Hinnen C. Attachment anxiety predicts poor adherence to dietary recommendations: an indirect effect on weight change 1 year after gastric bypass surgery. Obes Surg 2015; 25: 666–672. 25204408 10.1007/s11695-014-1423-7

[bib11] Burgmer R, Grigutsch K, Zipfel S, Wolf AM, de Zwaan M, Husemann B et al. The influence of eating behavior and eating pathology on weight loss after gastric restriction operations. Obes Surg 2005; 15: 684–691. 15946461 10.1381/0960892053923798

[bib12] Stunkard AJ, Messick S. The three-factor eating questionnaire to measure dietary restraint, disinhibition and hunger. J Psychosomc Res 1985; 29: 71–83. 10.1016/0022-3999(85)90010-8 3981480

[bib13] Zigmond AS, Snaith RP. The hospital anxiety and depression scale. Acta Psychiatr Scand 1983; 67: 361–370. 6880820 10.1111/j.1600-0447.1983.tb09716.x

[bib14] Fraley RC. Information on the Experiences in Close Relationships-Revised (ECR-R) Adult Attachment Questionnaire In: University of Illinois, 2012. Available at: http://internal.psychology.illinois.edu/~rcfraley/measures/ecrr.htm.

[bib15] Hayes AF. Introduction to Mediation, Moderation, and Conditional Process Analysis: A Regression-Based Approach. Guilford Press: New York, NY, USA, 2013.

[bib16] Hayes AF PROCESS: A versatile computational tool for observed variable mediation, moderation, and conditional process modeling. Available at: http://www.afhayes.com/public/process2012.pdf.

[bib17] Hays NP, Roberts SB. Aspects of eating behaviors “disinhibition” and “restraint” are related to weight gain and BMI in women. Obesity 2008; 16: 52–58. 18223612 10.1038/oby.2007.12 PMC2713727

[bib18] Suter M, Calmes JM, Paroz A, Giusti V. A 10-year experience with laparoscopic gastric banding for morbid obesity: high long-term complication and failure rates. Obes Surg 2006; 16: 829–835. 16839478 10.1381/096089206777822359

[bib19] Mikulincer M, Shaver PR. Attachment in Adulthood: Structure, Dynamics, and Change. Guilford Press: New York, NY, USA, 2007.

[bib20] Green J, Goldwyn R. Annotation: attachment disorganisation and psychopathology: new findings in attachment research and their potential implications for developmental psychopathology in childhood. J Child Psychol Psychiatry 2002; 43: 835–846. 12405473 10.1111/1469-7610.00102

[bib21] Sockalingam S, Wnuk S, Strimas R, Hawa R, Okrainec A. The association between attachment avoidance and quality of life in bariatric surgery candidates. Obes Facts 2011; 4: 456–460. 22248996 10.1159/000335345 PMC6444749

[bib22] Sockalingam S, Cassin S, Hawa R, Khan A, Wnuk S, Jackson T et al. Predictors of post-bariatric surgery appointment attendance: the role of relationship style. Obes Surg 2013; 23: 2026–2032. 23757051 10.1007/s11695-013-1009-9

[bib23] Mikulincer M, Shaver PR. Boosting attachment security to promote mental health, prosocial values, and inter-group tolerance. Psychol Inquiry 2007; 18: 139–156.

[bib24] Wilkinson LL, Rowe AC, Heath GH. Eating me up inside priming attachment security and anxiety, and their effects on snacking. J Soc Pers Relatsh 2013; 30: 795–804.

